# A Scaphoid Stress Fracture in a Female Collegiate-Level Shot-Putter and Review of the Literature

**DOI:** 10.1155/2016/8098657

**Published:** 2016-08-03

**Authors:** Jessica M. Kohring, Heather M. Curtiss, Andrew R. Tyser

**Affiliations:** ^1^Department of Orthopaedic Surgery, University of Utah, Salt Lake City, UT 84108, USA; ^2^Marshfield Clinic, Department of Sports Medicine, Physical Medicine & Rehabilitation, University of Wisconsin-Stevens Point, Marshfield, WI 54449, USA

## Abstract

Scaphoid stress fractures are rare injuries that have been described in young, high-level athletes who exhibit repetitive loading with the wrist in extension. We present a case of an occult scaphoid stress fracture in a 22-year-old female Division I collegiate shot-putter. She was successfully treated with immobilization in a thumb spica splint for 6 weeks. Loaded wrist extension activities can predispose certain high-level athletes to sustain scaphoid stress fractures, and a high index of suspicion in this patient population may aid prompt diagnosis and management of this rare injury.

## 1. Introduction

Scaphoid fractures are common in young adults and athletes and can lead to significant morbidity even with early diagnosis and appropriate treatment. While scaphoid fractures are most commonly associated with acute wrist trauma, it is notable that chronic repetitive loaded wrist extension can lead to scaphoid stress fractures [1].

Although rare, scaphoid stress fractures have been described in young, high-level athletes who exhibit repetitive loading with the wrist in extension, most commonly in gymnasts [2–7]. With increasing participation in high-level athletics at an earlier age, there has been a perceived increase in the incidence of pediatric and young adult stress fractures occurring in the upper extremity [5, 8]. Given the well-recognized challenges in diagnosing and managing scaphoid fractures, prompt recognition of these injuries—both acute and chronic varieties—is critical. Here we present a case of an occult scaphoid stress fracture in a 22-year-old female Division I collegiate shot-putter who was successfully treated nonsurgically and returned to sport.

## 2. Case Report

A 22-year-old female Division I collegiate right-hand dominant shot-putter initially presented with a two-month history of progressive, activity-related right wrist pain, with no report of prior trauma. She noted worsening pain with wrist extension during throwing the shot-put but had no complaints of pain or dysfunction with the discus nor with activities of daily living. The patient had been training for three to four hours per day, five times per week, alternating between the shot-put and discus as well as doing Olympic-style weight lifting for seven years prior to presentation.

**Figure 1:**
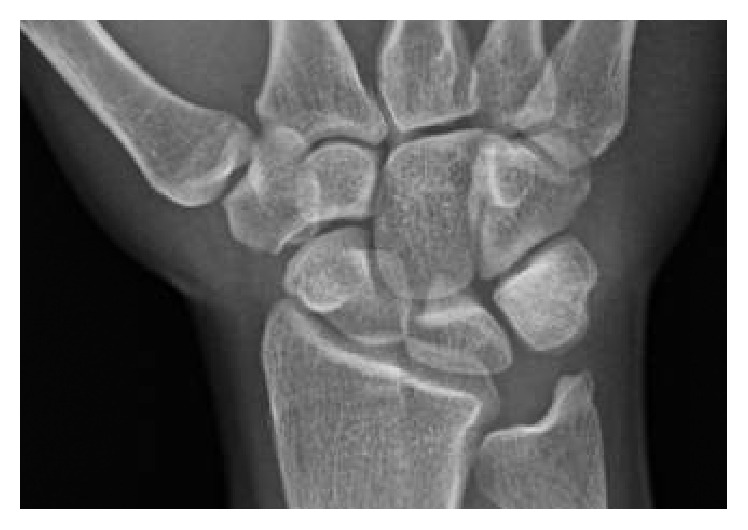
Posteroanterior radiographic view of the wrist at the time of initial evaluation that shows no abnormality in the scaphoid.

**Figure 2:**
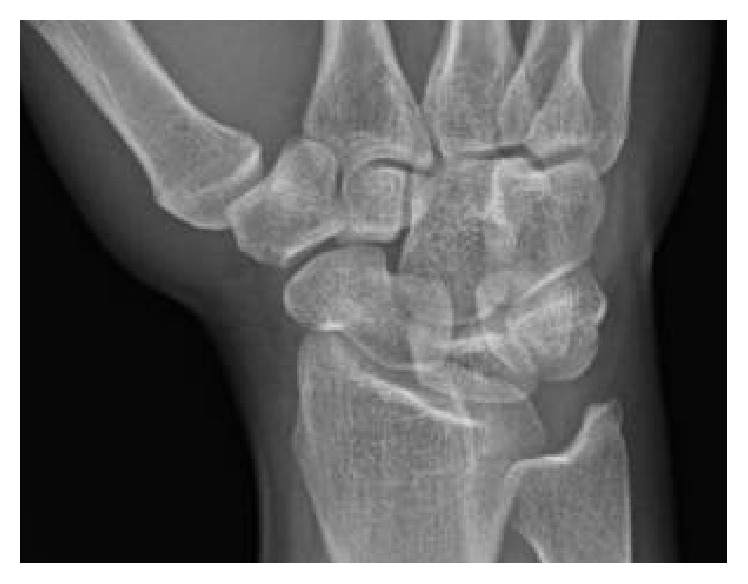
An oblique radiographic view at the time of initial evaluation without evidence of abnormality of the scaphoid.

**Figure 3:**
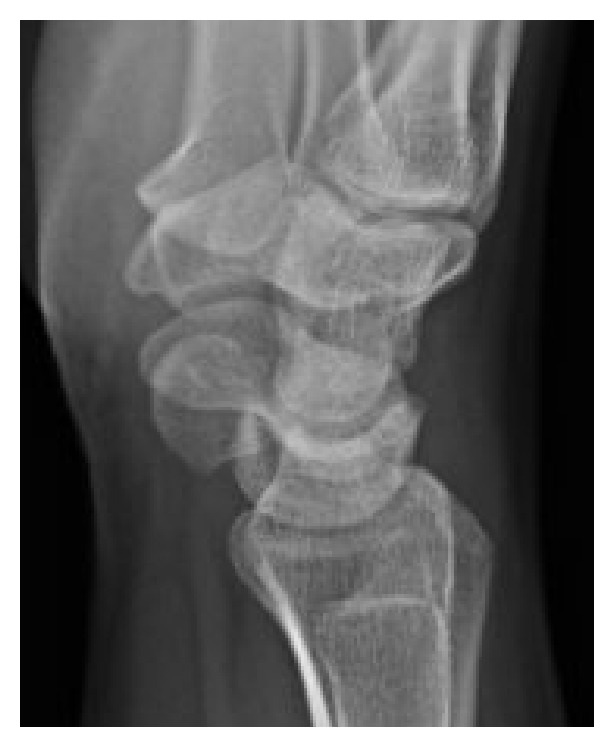
A lateral view of the wrist at the time of initial evaluation without radiographic abnormality of the scaphoid.

**Figure 4:**
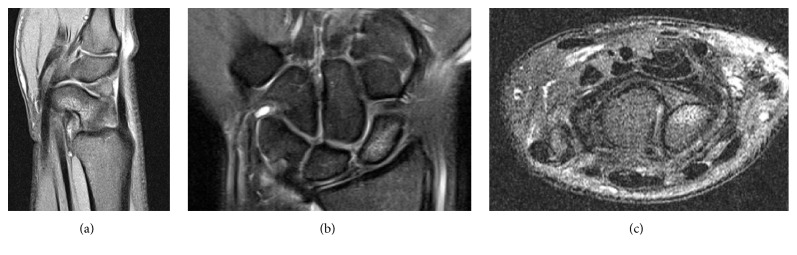
(a) A 1.5-Tesla MRI T2 sagittal cut demonstrating palmar scaphoid waist bone edema consistent with incomplete scaphoid waist stress fracture obtained at the time of initial presentation. (b) A 1.5-Tesla MRI T2 coronal cut showing scaphoid waist bone edema consistent with incomplete scaphoid waist stress fracture obtained at the time of initial presentation. (c) A 1.5-Tesla MRI T2 axial cut showing palmar scaphoid waist bone edema consistent with incomplete scaphoid waist stress fracture obtained at the time of initial presentation.

Physical exam revealed tenderness to palpation at the anatomic snuffbox and the scaphoid tuberosity. Wrist flexion, extension, and supination were symmetric, but painful with loaded terminal extension in the dominant wrist. Radiographs of the affected wrist at the time of presentation, demonstrated −1 mm ulnar negative variance, with no evidence of abnormality (Figures 1, 2, and 3). Due to a high level of suspicion, a noncontrast 1.5-Tesla MRI of the wrist was obtained. The MRI demonstrated an incomplete stress fracture at the scaphoid waist with associated bone edema and no cortical breakthrough, best seen on the T2 sagittal cut (Figure 4).

**Figure 5:**
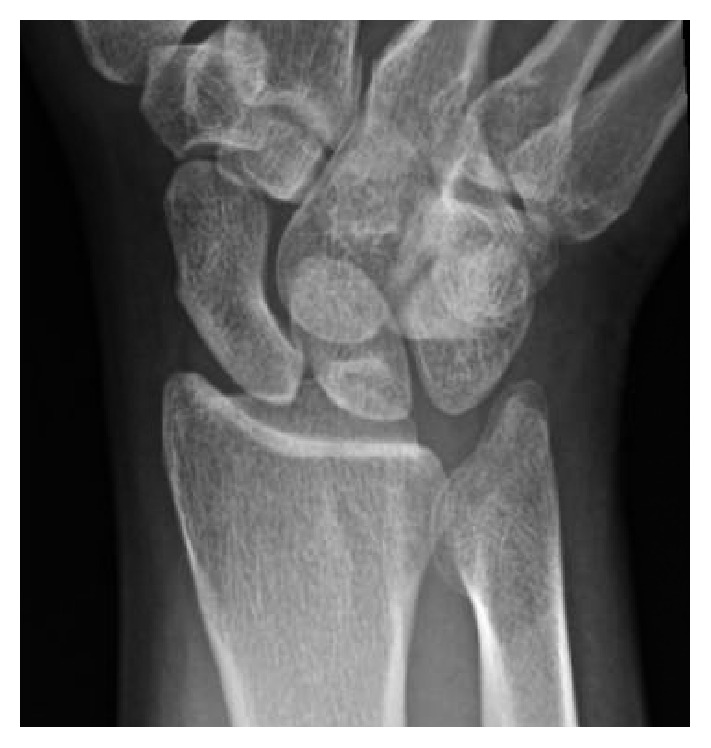
A scaphoid view obtained 6 weeks after presentation without radiographic evidence of a scaphoid waist fracture.

**Figure 6:**
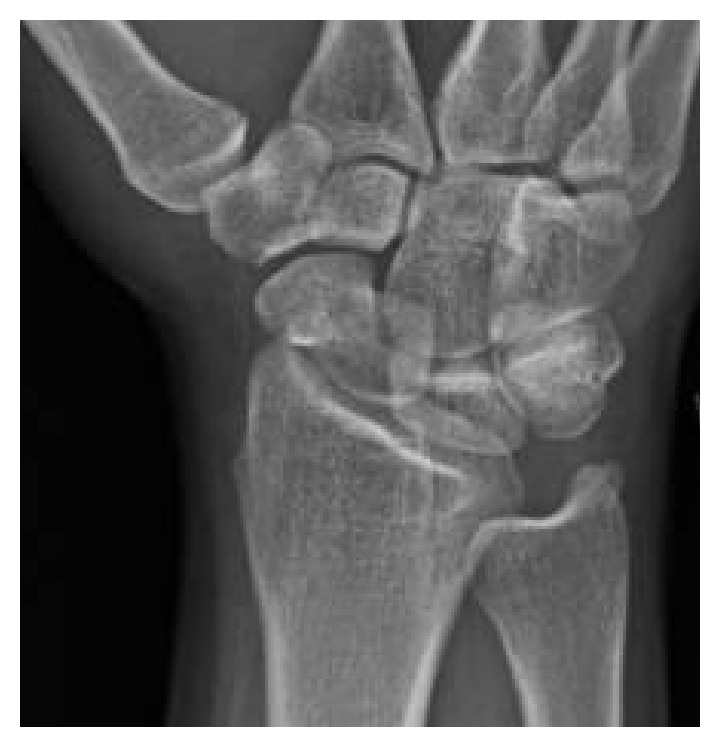
An oblique radiographic view obtained 6 weeks after presentation with no radiographic abnormality of the scaphoid.

**Figure 7:**
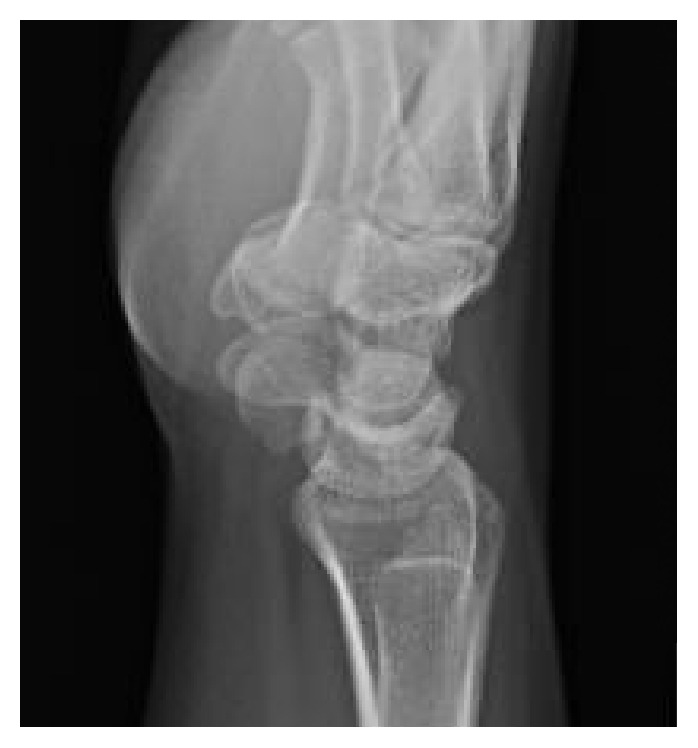
A lateral radiographic view obtained at 6 weeks after initial presentation showing a normal appearing scaphoid.

The patient was placed in a removable thumb spica wrist splint and was instructed to avoid any loaded extension of the wrist, including throwing the shot-put and weight-training. She was allowed to throw discus as it did not cause any pain. After three weeks, the patient reported no symptoms or pain with wrist extension. Radiographs obtained at 6 weeks after thumb spica immobilization were negative for any evidence of scaphoid fracture (Figures 5, 6, and 7). On physical exam, the patient had no tenderness to palpation in the anatomic snuffbox. The patient was released back to full activity without restrictions and returned to full participation in Division I shot-put without symptoms thereafter. At follow-up three years after her diagnosis, she reported no pain and no limitations in wrist or hand use.

## 3. Discussion

Scaphoid stress fractures are very rare injuries, with only case reports available for analysis in the peer-reviewed literature. In each reported case, the patients were competitive, high-level athletes training for multiple hours per day for several years prior to their presentation (Table 1). While gymnasts have been the athletes most commonly affected [2–7], others have also experienced these rare injuries: divers, soccer goalkeepers, shot-putters, badminton, cricket, and tennis players [3, 9–16].

Common to all of these athletic activities is the act of repetitive loaded wrist extension. Although the exact factors that lead to stress fractures of the scaphoid remain unclear, it has been suggested that repetitive stress and microtrauma to the bone can exceed native osseous repair mechanisms [3]. In each clinical case of a scaphoid stress fracture reported in the literature to date, including this one, the scaphoid waist was the location of the stress fracture.

Loaded wrist extension creates stresses that are typically centered at the scaphoid waist. In a cadaver study, Weber and Chao reported that 460 to 960 pounds of force applied to an extended wrist was required to acutely fracture the scaphoid, at the waist [17]. A more recent biomechanical study performed by Majima et al. found that loading the wrist in extension transmits force primarily through the scaphoid waist [18]. Handstands and other static maneuvers that require maximum wrist extension have been reported to exert considerable force across the scaphoid waist, but not to the extent needed to cause acute fracture [17].

Interestingly, the majority of scaphoid stress fractures have been reported in young male athletes, with 14 male and only 2 female patients reported in the available literature. The exact mechanism for this apparent gender discrepancy remains unclear but may be related to males reaching skeletal maturity at a later age than females, as adolescence appears to be a risk factor for suffering a scaphoid stress fracture. Similarly, while a direct link to age, sex, and athletic participation remains speculative, scaphoid stress fractures may be in part due to more intense participation in higher-level, longer-duration athletic training during adolescence.

Importantly, many of the published case reports regarding scaphoid stress fractures have noted a delay in diagnosis with this injury, with the majority of cases being recognized only after the fracture became apparent on plain radiographs [2, 3, 5]. Several case reports obtained bone scans to aid in their diagnosis of a scaphoid stress fracture [2–4], but more recently advanced imaging such as MRI and/or CTs has been utilized to diagnose or confirm scaphoid stress fractures [6, 7, 11, 13, 15, 16]. Several patients with negative presenting radiographs had the fracture only later diagnosed on repeat radiographs or advanced imaging [2, 3, 6]. In the case presented here, the presenting radiographs were negative, and an MRI was essential for making the diagnosis.

There are no current guidelines specific to the treatment of scaphoid stress fractures. However, for displaced or chronic scaphoid fractures or nonunions, surgical intervention is typically recommended. For nondisplaced or incomplete fractures, as in this case, nonsurgical treatment with immobilization is usually appropriate. Of the cases described in the literature, nine of the cases were treated nonoperatively [2, 3, 5, 9, 15, 16], two cases were initially treated nonoperatively but their patients had ongoing pain and evidence of nonunion requiring surgical intervention [6, 10], and five cases were treated with open reduction and internal fixation [7, 11–13]. For all patients treated with surgery, either a Herbert screw or a headless compression screw was used with the majority of cases also using bone autograft [6, 7, 10–13]. All patients reported had successful treatment outcomes regardless of the intervention with return to athletic activities and no reports of recurrence of pain, reinjury, nonunion, or malunion at longer-term follow-up.

In summary, stress fractures of the scaphoid are exceedingly rare but potentially devastating if not recognized and treated promptly. Clinicians should have a high index of suspicion when evaluating an athlete or patient who presents with an insidious onset of activity-related wrist pain and snuffbox tenderness and who is involved in a sport that requires repetitive loaded wrist extension. While the majority of cases described have involved male athletes, scaphoid stress fractures also occur in females. A low-threshold to obtain advanced imaging when radiographs appear negative for scaphoid pathology may aid in the early diagnosis of this rare entity, avoid fracture nonunion, and reduce the need for complex surgical intervention.

## Figures and Tables

**Table 1:** Clinical characteristics, imaging evaluation, and treatment method for scaphoid stress fractures published in the literature.

Author	Sport	Age (years)/ gender	Laterality	Pain duration	Time to diagnosis after presentation	Imaging presentation	Treatment	Scaphoid fracture location
Manzione and Pizzutillo [2]	Gymnast	16 M	Left	6 weeks	2 weeks	Negative XR; positive bone scan	Thumb spica cast ×10 weeks	Waist

Hanks et al. [3]	Shot-putter	19 M	Right	1.5 years	2 months	Negative initial XR; positive repeat XR at 2 months	Thumb spica cast/splint ×11 weeks	Waist
	Gymnast^*∗*^	18 M	Left	2 years	1 year, 2 weeks	Positive XR; positive bone scan prior to XR	Thumb spica cast ×4 months	Waist
			Right	3 weeks	No delay	Positive bone scan; negative XR	Thumb spica cast ×6 weeks	Waist
	Gymnast	18 M	Left	2 months	No delay	Positive bone scan; negative XR	Thumb spica cast ×6 weeks	Waist

Engel and Feldner-Busztin [4]	Gymnast	18 M	Bilateral	1 year	Not mentioned	Positive bone scan & XR	Not mentioned	Waist

Inagaki and Inoue [9]	Badminton	16 M	Right	7 weeks	No delay	Positive XR	Thumb spica cast ×8 weeks	Waist

Matzkin and Singer [5]	Gymnast	13 F	Right	3 months	3 months	Negative initial XR; positive XR 3 months later	Long arm spica ×8 weeks, short arm thumb spica ×4 weeks	Waist

Brutus and Chahidi [10]	Badminton	23 M	Right	8 weeks	No delay	Positive XR	Thumb spica cast ×8 weeks and then ORIF: Herbert screw & graft	Waist

Hosey et al. [11]	Diver	13 F	Right	2 months	No delay	Positive XR; confirmed on MRI	ORIF: Herbert screw	Waist

Rethnam et al. [12]	Cricketer	38 M	Right	2 years	No delay	Positive XR	ORIF: Herbert screw & graft	Waist

Yamagiwa et al. [6]	Gymnast	18 M	Right	Not mentioned	No delay	Positive MRI; negative XR	Thumb spica cast ×8 weeks and then ORIF: Herbert screw	Waist

Nakamoto et al. [7]	Gymnast	18 M	Right	3 months	No delay	Positive XR; confirmed on MRI	ORIF: Herbert screw	Waist

Pidemunt et al. [13]	Goalkeeper	13 M	Bilateral	2 years	No delay	Positive XR; confirmed on CT	ORIF: graft & Herbert screw	Waist

Mohamed Haflah et al. [14]	Diver	16 M	Bilateral	18 months (right)	1 year (right), no delay (left)	Positive XR R wrist (nonunion); incidental positive XR L wrist	ORIF: headless compression screw & graft	Waist

Saglam et al. [15]	Goalkeeper	19 M	Bilateral	4 years	No delay	Positive XR; confirmed on MRI	Thumb spica cast ×12 weeks	Waist

Kohyama et al. [16]	Tennis	18 M	Right	4 months	No delay	Positive XR; confirmed on CT & MRI	Thumb spica cast/splint ×12 weeks	Waist

Kohring et al. (current report)	Shot-putter	22 F	Right	2 months	No delay	Negative XR; positive MRI	Thumb spica splint ×6 weeks	Waist

^*∗*^Same patient with two different presentations.
